# In Vitro Anti-*Trypanosoma cruzi* Activity of Halophytes from Southern Portugal Reloaded: A Special Focus on Sea Fennel (*Crithmum maritimum* L.)

**DOI:** 10.3390/plants10112235

**Published:** 2021-10-20

**Authors:** Catarina G. Pereira, Carolina Borsoi Moraes, Caio H. Franco, Clarissa Feltrin, Raphaël Grougnet, Euzébio Guimarães Barbosa, Michele Panciera, Carlos Roque D. Correia, Maria João Rodrigues, Luísa Custódio

**Affiliations:** 1 Centre of Marine Sciences CCMAR, Faculty of Sciences and Technology, Ed. 7, Campus of Gambelas, University of Algarve, 8005-139 Faro, Portugal; cagpereira@ualg.pt (C.G.P.); mary_p@sapo.pt (M.J.R.); 2 Department of Microbiology, Institute of Biomedical Sciences, University of Sao Paulo, Sao Paulo 05508-900, SP, Brazil; cbmoraes@unifesp.br (C.B.M.); caiohaddadfranco@gmail.com (C.H.F.); clarissaufsc@gmail.com (C.F.); 3 Department of Pharmaceutical Sciences, Federal University of Sao Paulo, Diadema 09913-030, SP, Brazil; 4 Natural Products, Analysis, Synthesis, UMR CNRS 8038, Faculty of Pharmacy, University of Paris, 4 Avenue de l’Observatoire, 75006 Paris, France; raphael.grougnet@yahoo.fr; 5 Department of Pharmacy, Universidade Federal do Rio Grande do Norte, Natal 59064-720, RN, Brazil; euzebiogb@gmail.com; 6 Institute of Chemistry, State University of Campinas, Josue de Castro St., Campinas 13083-970, SP, Brazil; michele2707@hotmail.com (M.P.); roque@iqm.unicamp.br (C.R.D.C.)

**Keywords:** Marine halophytes, Chagas disease, *Trypanosoma cruzi*, neglected tropical diseases, *Crithmum maritimum*, falcarindiol

## Abstract

Marine halophytes are an outstanding reservoir of natural products and several species have anti-infectious traditional uses. However, reports about their potential use against neglected tropical ailments, such as Chagas disease, are scarce. This work evaluated for the first time the in vitro anti-*Trypanosoma cruzi* activity of extracts from the aromatic and medicinal species *Helichrysum italicum* subsp. *picardii* (Boiss. & Reut.) Franco (Asteraceae, everlasting) and *Crithmum maritimum* L. (Apiaceae, sea fennel). For that purpose, decoctions, tinctures, and essential oils from everlasting’s flowers and sea fennel’s stems, leaves, and flowers were tested against intracellular amastigotes of two *T. cruzi* strains. The extract from the sea fennel flower decoction displayed significant anti-trypanosomal activity and no toxicity towards the host cell (EC_50_ = 17.7 µg/mL, selectivity index > 5.65). Subsequent fractionation of this extract afforded 5 fractions that were re-tested in the same model of anti-parasitic activity. Fraction **1** was the most active and selective (EC_50_ = 0.47 μg/mL, selectivity index = 59.6) and was submitted to preparative thin-layer chromatography. One major compound was identified, falcarindiol, which was likely the one responsible for the observed anti-trypanosomal activity. This was confirmed using a commercially sourced molecule. Target-fishing studies showed falcarindiol as a ligand of *T. cruzi* spermidine synthase, pointing to a potential enzyme-inhibiting anti-trypanosomal mechanism of action. Overall, this work shows that sea fennel can provide effective anti-parasitic molecule(s) with potential pharmacological applications in the treatment of CD.

## 1. Introduction

Neglected tropical diseases (NTDs) are a group of disabling and chronic infections that flourish primarily in impoverished environments impairing the lives of over one billion people worldwide [1,2]. Among them is Chagas disease (CD), a potentially life-threatening zoonosis caused by the protozoan *Trypanosoma cruzi*. CD is mainly vector-borne, transmitted to humans through contact with the faeces/urine of triatomine bugs (kissing bugs), but can also occur by ingestion of contaminated foods, congenital transmission (mother to foetus), and blood transfusion or organ transplants. Traditionally confined to Central and South America, CD is a health and socioeconomic burden that has spread and is now an emergent global epidemic, with around eight million people infected worldwide [3,4,5,6]. CD involves acute and chronic phases, being more often diagnosed in the chronic stage as the acute infection is typically asymptomatic. In the acute phase, trypomastigotes circulate in the blood (parasitaemia) and infect cells, where they transform into asexually multiplying amastigotes. When the amastigote-containing cell is broken, parasites are released and infect other cells. An intense inflammatory response with activation of the innate immune response controls the parasite and after four to eight weeks parasitaemia decreases substantially. The acute stage usually resolves spontaneously leaving patients chronically infected, if untreated. In the chronic phase, parasites reach and establish in target organs, forming amastigote nests. This stage progresses slowly, and most chronic patients show no further signs of the disease. After several years, 30–40% of chronic patients will develop potentially fatal organ involvement (cardiomyopathy, megaviscera). Recent evidence shows that tissue damage is a result from *T. cruzi* action and the chronic inflammatory response it elicits [4,5,6,7]. Currently only two anti-parasitic drugs are approved for CD treatment, namely nifurtimox and benznidazole, and their efficacy, although high at the acute stage onset, is low in the chronic phase. Moreover, they are rather toxic, showing several side effects, and require prolonged administration [3,4,6,7,8,9]. Therefore, the development of new, effective, safe, and affordable drugs for CD remains an urgent need [4,5]. 

Marine halophytes, a specialized group of plants able to thrive in saline environments, have evolved several adaptations in response to the osmotic and ionic challenges of living in such harsh conditions, including the synthesis of highly bioactive metabolites. They represent an outstanding reservoir of natural compounds with some species being used in folk medicine as anti-parasitic and anti-helminthic [10]. However, reports on their potential use against NTDs like CD are scarce [11,12,13]. *Crithmum maritimum* L. (Apiaceae, sea fennel) and *Helichrysum italicum* (Roth) G. Don subsp. *picardii* (Boiss & Reuter) Franco (Asteraceae, everlasting) are two aromatic halophytes with described anti-infective uses, namely anti-helminthic and anti-mycotic, and validated antibacterial activities [14,15]. In this context, this work evaluated for the first time the in vitro properties of decoctions, tinctures, and essential oils (EOs) from both halophytes (following the usage given in folk practices) against the intracellular amastigotes of two *T. cruzi* strains. Preparative thin-layer chromatography (TLC) followed by nuclear magnetic resonance (NMR) analysis was used to identify the major compound likely responsible for the anti-trypanosomal activity and target fishing studies were performed to uncover probable mechanisms of action.

## 2. Materials and Methods

### 2.1. Chemicals

All chemicals were of analytical grade. Culture media were purchased from Welgene, Inc. (Seoul, South Korea), fetal bovine serum (FBS) and penicillin/streptomycin from Gibco Inc. (Life Technologies, Billings, MT, USA), DRAQ5^TM^ from BioStatus Ltd. (Loughborough, UK), benznidazole from Epichem Pty Ltd. (Perth, Australia), and falcarindiol from ChemSpace US Inc. (Monmouth Junction, NJ, USA). Additional reagents/solvents were obtained from VWR International (Leuven, Belgium).

### 2.2. Sample Collection 

Specimens of *Helichrysum italicum* (Roth) G. Don subsp. *picardii* (Boiss & Reuter) Franco (everlasting, Asteraceae family; voucher code XBH32) were collected in Ria Formosa coastal lagoon (37°01′54.0″ N 8°02′12.1″ W), south Portugal, in July 2015. *Crithmum maritimum* L. (sea fennel, Apiaceae family; voucher code XBH33) was collected from Aljezur beach (37°20′30.7″ N 8°51′06.0″ W), south Portugal, in August of 2015. Botanist Dr. Manuel J. Pinto (National Museum of Natural History, University of Lisbon, Botanical Garden, Portugal) performed the taxonomical classification. Voucher specimens are kept in the herbarium of XtremeBio’s laboratory (CCMAR, University of Algarve, Portugal). Sea fennel plants were divided into stems, leaves, and flowers, while only flowers from the everlasting were used. Plant material was oven-dried for 3 days at 40 °C until complete dryness, then powdered and stored at −20 °C until needed.

### 2.3. Preparation of the Extracts

Water extracts were prepared similarly to decoctions, by boiling 1 g of dried biomass for 5 min in 200 mL of ultrapure water. Hydro-ethanolic extracts were prepared similarly to tinctures, by homogenizing 20 g of dried biomass in 200 mL of 80% aqueous ethanol for a week. Extracts were filtered (Whatman n° 4), vacuum and/or freeze-dried, and stored in a cool, dark, and moisture-free environment. To obtain the essential oils (EOs), fresh biomass (500–1000 g, depending on biomass availability) was cut into small pieces and subjected to hydro-distillation in a Clevenger-type apparatus for 3 h; EOs were dried with sodium sulphate, filtered, weighed, and stored in sealed glass vials at −20 °C until further use.

### 2.4. Fractionation of the Active Extract

After a primary screening of the extracts’ anti-trypanosomal activity (described in Section 2.5), the active extract, sea fennel’s decoction from flowers, was fractionated: a 500 mL decoction was prepared and subjected to a sequential liquid–liquid partition using solvents of increasing polarity (hexane, dichloromethane, chloroform, and ethyl acetate, 150 mL each; fractions **1** to **4**, respectively). All fractions, including the water residue (fraction **5**), were vacuum concentrated and/or freeze-dried and stored until assessment for anti-trypanosomal activity in a secondary screening (described in Section 2.5).

### 2.5. Evaluation of In Vitro Anti-Trypanosomal Activity

All mammalian cell lines, namely human osteosarcoma, U2OS, and *Macaca mulatta* kidney epithelial, LLC-MK2, cells, previously available in C.B. Moraes laboratory, were cultured in DMEM medium supplemented with 10% heat-inactivated FBS, 100 U/mL penicillin, and 100 mg/mL streptomycin in a humid atmosphere (5% CO_2_, 37 °C). LLC-MK2 cultures maintained the *T. cruzi* mammalian cycle in vitro and these tissue-derived trypomastigote forms were used to infect U2OS cells in the anti-trypanosomal assay. Two *T. cruzi* strains corresponding to two of the six discrete typing units (DTUs; Sylvio X10/1 strain, DTU I; Y strain, DTU II) were chosen pragmatically based on stocks available at the start of the study. In vitro culture of *T. cruzi* was performed as previously described [16]. 

Extracts or fractions were dissolved in DMSO either at 5, 10, or 20 mg/mL (according to the different saturation points), the positive control compound benznidazole was dissolved at 40 mM in DMSO, and the compound falcarindiol at 20 mM in DMSO. The anti-trypanosomal assays were performed in duplicate (2 independent experiments), following Moraes et al. [16]; plates were fixed, and parasite and host cell DNA were stained with DRAQ5^TM^ for microscope imaging (high content screening imaging system, Operetta, Perkin Elmer). A primary single-concentration screening was carried out with the extracts (100 μg/mL final concentration, 200 μM for benznidazole) to assess normalized activity (percentage of infection ratio reduction) and average cell ratio (extracts’ cytotoxicity). The active extract, and afterwards fractions **1** to **5** (see Section 2.4), were subjected to a secondary confirmatory dose-response screening, following a 2-fold serial dilution (10 points, 100 μg/mL as the highest concentration tested), with *T. cruzi* Y strain (the only strain yielding results for the active extract). The commercial compound falcarindiol was tested in concentration-response against the Y strain (clone H10) [17] following the same assay protocol described above.

#### Data Analysis

Acquired images were analyzed with high content analysis software (Harmony, Perkin Elmer) to detect host cell cytoplasm boundary, host cell nucleus, and *T. cruzi* DNA, which in turn were quantified to determine total number of cells, number of infected cells, ratio of infected cells, and average number of parasites per infected cell. Only intracellular parasites were scored. Values for ratio of infected cells (infection ratio) were normalized to the average ratio of infected cells from all negative (infected, non-treated cells) and positive (non-infected cells) controls to obtain normalized activity/antiparasitic activity. Average cell ratio was determined by the ratio between total cell number in a test well and average total cell number in negative control wells. Cell ratio was determined against infected controls since *T. cruzi* infection can also reduce cell numbers due to a cytolytic effect resulting from parasite release from infected cells and, thus, comparison to infected controls is more accurate to determine the contribution of compound cytotoxicity to the reduction in cell number. Normalized activity datasets were fitted in dose-response curves using GraphPad Prism^®^ to determine EC_50_ (concentration that reduces the infection in 50%), CC_50_ (concentration that reduces the number of cells in 50%), selectivity index (CC_50_/EC_50_), and maximum activity (max. infection inhibition). Data analysis is described in detail in Moraes et al. [16].

### 2.6. Chemical Analysis

An amount of 50 mg of the active and selective fraction **1** obtained as described in Section 2.4 was submitted to preparative thin-layer chromatography (TLC), using ethyl acetate/hexane 3/7 as the eluent. After UV light (254 nm) examination and careful spraying of the TLC sides with sulfuric vanillin, the five evidenced bands were removed from the plate and extracted from the silica by ultrasonication for 30 min in dichloromethane. After filtration and evaporation under reduced pressure, the compound in higher quantity (mg), coincidentally corresponding to the major band, was chosen and the residue was dissolved in 0.5 mL of deuterated chloroform. Nuclear magnetic resonance (NMR) experiments were carried out using a Bruker 400 MHz Avance spectrometer and Bruker pulse programs for data acquisition. Results were examined using Mnova software version 6.0.2.

### 2.7. Target Fishing Hypothesis

An inverse ligand-based virtual screening search was carried out to hypothesize which targets might be involved in the anti-*T. cruzi* activity. The Ligand Expo data bank (ligand-expo.rcsb.org) was the library used to perform similarity searches using falcarindiol as the reference compound. Around 32,000 compounds in their biologically active conformations were compared to falcarindiol using the pharmaACOphore multiple flexible ligand alignments based on ant colony optimization (ACO) [18]. Similarity scores were obtained using ShaEP, which performs rigid-body superimposition of the aligned 3D molecular models [19]. The top 300 similarity scores were selected and filtered. Eukaryotic molecular targets were selected for an additional structures-based search (see Tables S1 and S2 in Supplementary Materials). The Autodock vina software allowed docking falcarindiol to the binding sites and gave the scoring function along with the binding poses, providing refined results. The most reasonable target and the searched ligand, along with the adjacent binding sites’ conformations, were optimized using the UCSF Chimera software (minimize structure tool) [20]. Docking results were further optimized using GROMACS [21], employing the GROMOS 54a7 force field [22]. Ligand topologies for the same force field were derived from the ATP server [23]. The solvent TIP3P water model was employed in a charge-neutralized simulation box. Geometry optimization of the solvated system was performed using the steepest descent algorithm. The system was equilibrated using nVT and nPT ensembles with the protein kept fixed. Unrestrained molecular dynamics simulation was performed for 10 ns, enough to ensure protein backbone RMSd (root-mean-square deviation) stabilization. The ligand stability during the simulations was analyzed by calculating its RMS deviations.

## 3. Results

Dried biomass was extracted with water (decoctions) and 80% aqueous ethanol (tinctures) while fresh biomass was used to extract EOs, resulting in higher yields (Table 1) for sea fennel’s water extracts, particularly leaves (45.7%) and flowers (37.8%), and for everlasting’s tinctures (32.6%). As expected, EO yields were lower, reaching 0.53% for sea fennel’s flowers.

To assess the in vitro anti-trypanosomal properties of sea fennel and everlasting samples (decoctions, tinctures, and EOs) against intracellular amastigotes of two *T. cruzi* strains, a primary single-concentration screening was carried out. Results for normalized activity (Table 1), which gives the percentage of infection inhibition in relation to controls, pointed to 5 promising extracts with ≥50% activity against the *T. cruzi* Y strain and 4 extracts against the *T. cruzi* Sylvio X10/1 strain. However, when cross-checking with average cell ratio (Table 1), an indicator of extracts’ cytotoxicity toward host cells that should desirably be ≥0.5, only the sea fennel flower decoction fulfilled both criteria and only for the Y strain (65% activity, 0.73 cell ratio). This active extract was subjected to a confirmatory dose-response screening with the *T. cruzi* Y strain and its anti-parasitic activity was corroborated (Table 2): an EC_50_ of 17.7 μg/mL and almost 90% maximum activity indicate potency and efficacy against the parasite, and with no toxicity towards the host cell detected within the tested concentrations (if CC_50_ is not determined, the highest tested concentration is used to estimate the SI). Being the most active and least cytotoxic extract, the sea fennel flower decoction was fractionated by liquid–liquid partition using hexane, dichloromethane, chloroform, and ethyl acetate, and all fractions (including the water residue) were evaluated for anti-trypanosomal activity against the *T. cruzi* Y strain. Results (Table 3) show that the hexane fraction (fraction **1**) was the most active and selective, presenting higher potency (EC_50_ = 0.47 µg/mL) and efficacy (113% max. activity), the lowest cytotoxicity (CC_50_ = 28.0 µg/mL), and higher selectivity towards the host cells (SI = 59.6). The positive control benznidazole had comparatively lower potency (EC_50_ = 0.92 µg/mL) while similar efficacy and similar or higher selectivity (109% max. activity, SI > 56). In addition, fraction **2** (dichloromethane) exhibited high efficacy against the parasite (97% max. activity) and moderate selectivity (SI > 6.47), with an EC_50_ slightly lower and therefore more potent than that of the crude extract (fraction **2**, EC_50_ = 12.3 µg/mL, Table 3; sea fennel flower decoction, EC_50_ = 17.7 µg/mL, Table 2).

The active and selective hexane fraction **1** was submitted to preparative TLC to recover and identify the major metabolite. Its molecular formula C_17_H_24_O_2_ was deduced from the protonated molecule observed on ESI-MS at m/z = 261.3, indicating 6 degrees of unsaturation. The ^1^H and ^1^H-^13^C 2D HSQCed NMR spectra showed the signals of seven deshielded protons, including five olefinic carbons and two hydroxylated ones, and of a saturated alkyl moiety comprising a methyl group and methylene protons. Examination of ^13^C and ^1^H-^13^C 2D HMBC NMR spectra discriminated four acetylenic positions (see spectra in Figure S3 Supplementary Materials). All these data are in agreement with the chemical shifts and structure previously reported for falcarindiol [24]. This compound was already characterized in sea fennel [25]. 

To confirm that the major compound identified, falcarindiol, was responsible for the observed anti-trypanosomal activity, the molecule was commercially sourced and tested against the *T. cruzi* Y strain. Results (Table 4) confirm that falcarindiol is active, showing an EC_50_ comparable (6.8 µM; 1.77 µg/mL) to that of the active fraction **1** (EC_50_ = 0.47 µg/mL; Table 3). Falcarindiol was effective in vitro, reducing *T. cruzi* infection to undetectable levels (maximum activity greater than 100%) while it was not cytotoxic up to 100 µM (26 µg/mL), presenting similar values to those obtained with active fraction **1**. Falcarindiol was also as effective and slightly more potent than benznidazole under the tested conditions.

**Figure 1:**
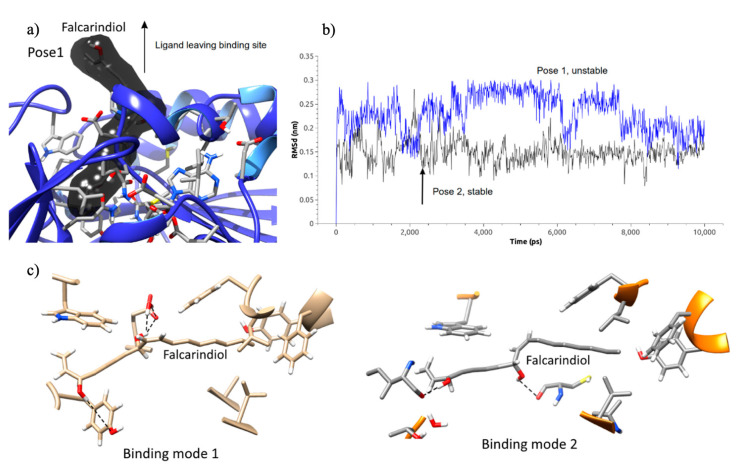
(**a**) Unstable binding pose derived from molecular docking where the ligand did not hold inside the initial position; (**b**) RMSd from the initial ligand positions showing extensive variation for one of the poses (pose 1, unstable) and quicker stabilization when falcarindiol had its hydroxyl groups buried deeper (pose 2, stable); (**c**) two alternating sets of H-bond interactions between falcarindiol and surrounding amino acid residues.

Possible targets involved in the anti-*T. cruzi* activity of the active molecule falcarindiol were inferred by performing an inverse ligand-based virtual screening procedure. Similarity searches with falcarindiol structure resulted in good scores (near 1.0) with lipid-like compounds not related to the targets’ activity. Spermidine (PDB Chemical ID: SPM; Table S1 in Supplementary Materials) was as elongated as falcarindiol with similar pharmacophoric points and was related to the targets’ mechanism; hence, it was considered a reasonable candidate for further in silico studies. In this sense, the crystal structure of spermidine synthase from *Plasmodium falciparum* in complex with spermine (10.2210/pdb3B7P/pdb) was a reasonable hypothetical target. Falcarindiol and spermidine possess similar molecular volume, shape, and polarity, proving to be a reasonable compatible fit. A homology model for the *T. cruzi* homologue sequence (GenBank: PBJ69308.1) with 44.13% identity (e-value: 9e−77, 94% cover) was built using the MODELLER software [26] to carry out the falcarindiol binding molecular docking and optimization procedures. The model created has very high-quality indications despite the lower level of identity, with PDB 3B7P (*Plasmodium falciparum*) and 4YUV (*Trypanosoma cruzi*) and the model being quite similar. However, the structural approach was refined by two molecular dynamics simulations to optimize the homology models and spermidine synthase-falcarindiol interactions. Two binding poses with the most negative docking scores were used as a starting point. One of the initial falcarindiol binding poses was unstable (pose 1) and the ligand escaped the interaction’s site driven by the surrounding solvent (Figure 1a). During simulation, RMSd (root-mean-square deviations) from the initial ligand positions varied extensively for one of the poses (pose 1; Figure 1b). Falcarindiol’s most stable binding pose (pose 2) was the one where falcarindiol kept its hydroxyl groups buried deeper in the spermine site and was able to stabilize faster during the simulation (Figure 1b) and form two hydrogen bonds with adjacent residues (Figure 1c). Two alternating sets of H-bond interactions were formed between falcarindiol and backbone carbonyl moieties or surrounding amino acid residues (TYR and GLU residues; Figure 1c). The obtained interaction strengthens the hypothesis that spermidine synthase could be related to the observed anti-trypanosomal activity of falcarindiol against *T. cruzi*.

## 4. Discussion

Current anti-parasitic treatment for CD relies on the drugs benznidazole and nifurtimox, both associated with severe side effects and debatable efficacy in the chronic phase, which highlights the need to find novel anti-trypanosomal therapies [4,6,7]. Recent efforts include improvement of current treatments, like combining benznidazole with other compounds or dosing adjustments, molecular targeted drug development, repositioning of known drugs, and discovery of novel compounds, like metal–drug complexes, chemically modified nitro-aromatic molecules, or plant-derived products [7,27]. However, despite the many promising documented drugs, others are needed due to the slow and rigorous validation process and high downstream failure of drug candidates [7,16]. For example, ravuconazole (E1224) and posaconazole were promising new drugs to treat chronic CD that were unsuccessful in human trials due to the absence of prolonged effects [28,29].

Plants represent an immense source of potentially bioactive molecules with anti-infectious activity including against *T. cruzi*, as for example rosemary (*Rosmarinus officinalis* L.) or green tea (*Camellia sinensis* (L.) Kuntze) [7], to name a few. Quite recently, some Amaryllidaceae alkaloids have been shown to inhibit *T. cruzi* growth, particularly hippeastrine, which was selective and specific against *T. cruzi* amastigotes (IC50 = 3.31 μM) [30]. However, halophytes have been overlooked as prospective sources of anti-protozoal compounds, especially against *T. cruzi*. To the best of our knowledge, only Oliveira et al. [12] screened several halophytes for in vitro anti-trypanosomal activity, finding one extract from *Juncus acutus* L. roots able to decrease *T. cruzi*’s growth, while López et al. [11] found that α-amyrine and quercetin isolated from the mangrove plant *Pelliciera rhizophorae* Planch. & Triana were active against *T. cruzi*. No reports were found in literature concerning the potential anti-parasitic activity of sea fennel and everlasting towards *T. cruzi*, although aerial parts, including flowers, have reported anti-infective medicinal uses [14,15]. In this context, this work evaluated for the first time the in vitro anti-trypanosomal activity of decoctions, tinctures, and essential oils (following the usage given in folk practices) from those aromatic halophytes against intracellular amastigotes of two *T. cruzi* strains.

Most of the tested samples did not yield promising anti-chagasic activity, either by low efficacy or due to high host cell toxicity, particularly when compared to reference compound benznidazole (200 µM final concentration; Table 1). The exception was the decoction from sea fennel’s flowers that displayed moderate activity with 65% infection reduction without significantly affecting the host cell. However, these results were obtained for the Y strain only, probably due to the Sylvio X10/1 strain’s higher infectivity and superior number of intracellular amastigotes. Despite presenting high genetic similarity, *T. cruzi* strains yield distinct susceptibility to different compounds, depending on the target [31]. For instance, the activity of ergosterol biosynthesis inhibitors (posaconazole, ravuconazole, and others) varied greatly depending on the *T. cruzi* strain assayed in vitro, under the same assay conditions [16]. Even for reference antichagasic compounds, such as benznidazole and nifurtimox, the in vitro activity is expected to vary between Y and Sylvio strains, which might be influenced by distinct infectivity profile-cellular invasion and differentiation capacities and intracellular multiplication [32,33]. The confirmatory dose-response screening of the active extract with the *T. cruzi* Y strain corroborated its anti-parasitic activity (EC_50_ = 17.7 μg/mL) and with no toxicity detected towards the host cells (Table 2). Subsequent fractionation of the sea fennel flower decoction and assessment of anti-trypanosomal activity in the resulting 5 fractions showed the hexane fraction (fraction **1**) as the most active (EC_50_ = 0.47 μg/mL) and selective, and fraction **2** (dichloromethane) with a residual effect (EC_50_ = 12.3 μg/mL) (Table 3).

One major metabolite was identified in fraction **1**, falcarindiol, which was likely the one responsible for the anti-trypanosomal activity. Considering falcarindiol’s structure, it would have been easily extracted from the aqueous phase by hexane, while a small proportion probably remained in the decoction and was afterwards removed by dichloromethane, potentially accounting, at least partly, for the biological effect of fraction **2** (Table 3). Further testing against the *T. cruzi* Y strain confirmed the anti-trypanosomal activity of falcarindiol, with similar potency (EC_50_ = 6.8 µM; 1.77 µg/mL; Table 4) to that of fraction **1** (EC_50_ = 0.47 µg/mL; Table 3). No cytotoxicity was detected for falcarindiol up to 100 µM (26 µg/mL), similarly to fraction **1** (CC_50_ = 28 µg/mL), while it effectively reduced *T. cruzi* infection to undetectable levels (maximum activity higher than 100%, like for fraction **1**), thus demonstrating that this molecule is highly selective towards *T. cruzi* amastigotes. In the only studies available on falcarindiol’s trypanocidal effects, Salm et al. [34] reports that the polyacetylene isolated from *Sium sisarum* L. had no inhibitory effect on *T. cruzi*, while Mennai et al. [35] describes a low anti-trypanosomal activity of this constituent identified in *Pituranthos battandieri* Maire. Nevertheless, the former performed antiproliferation assays on *T. cruzi* epimastigotes (IC_50_ > 50 µM) and trypomastigotes (0% parasite release inhibition at 5 µM), and the latter assayed on epimastigote forms of *T. cruzi* (IC_50_ = 121.8 µM). The present work performed anti-trypanosomal screenings against the intracellular amastigote form since it better represents the *T. cruzi* tissue infection leading to CD and it is the main parasite form in the chronic stage [4,36]. The use of different morphological forms of the parasite may explain the divergent reports on the anti-*T. cruzi* activity of falcarindiol, as compounds can present disparate activity against trypomastigotes, intracellular amastigotes, and epimastigotes [27]. Despite variations in falcarindiol’s activity being potentially due to the different life stages of *T. cruzi*, the concentration could also account for the different results: falcarindiol was only active against epimastigotes at high concentrations (>50 µM) [34,35], and only a low concentration (5 µM) was tested against trypomastigotes in the release assay [34].

**Figure 2:**
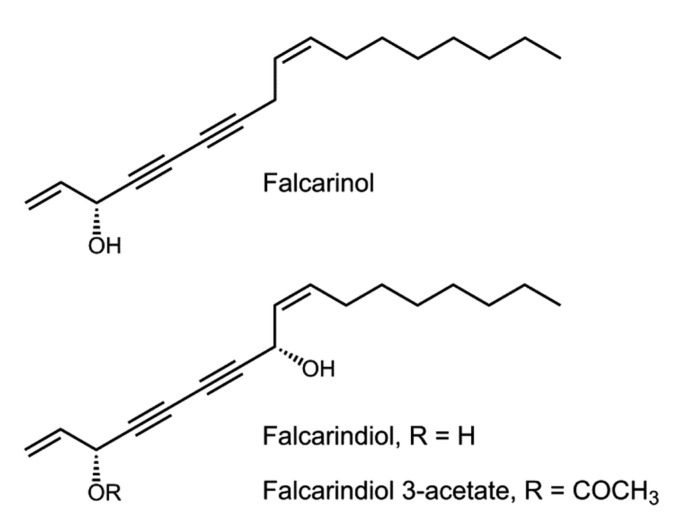
Chemical structures of (3R)-falcarinol (FaOH), (3R,8S)-falcarindiol (FaDOH), and (3R,8S)-falcarindiol 3-acetate (FaDOH3Ac) (adapted from Kobaek-Larsen et al. [42]).

Another structurally related C_17_-polyacetylene, falcarinol (also known as panaxynol), has already been described as a main compound in sea fennel’s leaves [37] and has also been reported as toxic (EC_50_ = 0.01 μg/mL) and highly selective against another *Trypanosoma* species, *T. b. brucei*, the parasite causing Human African Trypanosomiasis [38]. Aliphatic C17-polyacetylenes of the falcarinol-type such as falcarinol and falcarindiol (Figure 2) have shown many interesting bioactivities (antifungal, neurotoxic, cytotoxic, allergenic, anti-inflammatory, anti-platelet-aggregatory, antibacterial) and, although their exact mechanisms of action are not entirely known, they seem to be related to their alkylating properties, namely enzyme inhibition via covalent alkylation, and hence their ability to interact with various biomolecules [39,40]. Moreover, monoterpenes that can be found in sea fennel’s EOs, such as α-pinene, β-ocimene, limonene, and sabinene, have been described as active and selective against *T. brucei* [13], while a less abundant monoterpene found in sea fennel, linalool [14], showed a potent trypanocidal effect against *T. cruzi* trypomastigotes (IC_50_ = 0.31 μg/mL) [41]. Fraction **1**, besides the identified compound falcarindiol, could also contain some of the above-mentioned essential oil components with reported anti-parasitic effects, considering that a decoction (hot water) may allow extraction of such apolar metabolites in low proportions. During liquid–liquid partition these compounds would logically concentrate in the organic phase, leading to a hexane-enriched fraction, and could eventually be at play in the anti-*T. cruzi* activity of the fraction.

Monoterpenes and polyacetylenes represent classes of secondary metabolites with promising lead compounds to develop novel trypanocidal drugs [13]. To affect the intracellular amastigote form of the parasite, compounds must be able to pass through the host-cell’s plasma membrane [43]. Several monoterpenes and polyacetylenes are lipophilic and can therefore cross the plasma membrane and disturb biomembranes within the cell [44]; monoterpenes, in particular, can cause destabilization of the protozoal plasma membrane and/or cause cell lysis [45]. However, the target fishing studies currently performed showed the active molecule falcarindiol as a ligand of *T. cruzi* spermidine synthase, suggesting an enzyme-inhibiting anti-trypanosomal mechanism of action. The observed activity could even arise from a synergistic action of the polyacetylene falcarindiol inhibiting a key-enzyme and apolar monoterpenes destabilizing the parasite membrane.

Overall, literature shows that there are many secondary plant metabolites that can have anti-trypanocidal activity and medicinal plants in particular, like sea fennel in the present study, can provide effective anti-parasitic molecules. In fact, our results indicate that falcarindiol identified in the active fraction **1** is responsible for its anti-trypanosomal activity, underlining the potential of polyacetylenes as lead compounds to develop novel trypanocidal drugs. The importance of identifying new trypanocidal compounds lies in the possibility of using them as novel or integrative therapies in CD treatment and/or as the starting material for new drug design.

## 5. Conclusions

To the best of our knowledge, this is the first report of sea fennel’s in vitro anti-*Trypanosoma cruzi* activity. A decoction of its flowers showed activity against *T. cruzi* intracellular amastigotes with no toxicity towards the host cells; the anti-trypanosomal properties resided in the most apolar fraction **1**. One major compound was identified, falcarindiol, subsequently confirmed as responsible for the anti-trypanosomal activity. Further research could attest for the proposed mode of action and decipher structure–activity relationships (SARs) to unravel pharmacological applications of this molecule. Overall, this work shows that sea fennel can provide effective anti-parasitic molecule(s) with potential pharmacological application in the treatment of CD.

## Figures and Tables

**Table 1:** Extraction yields and primary screening of anti-parasitic activity of *C. maritimum* and *H. italicum* subsp. *picardii* extracts against two *T. cruzi* strains. Normalized activity indicates infection inhibition and average cell ratio indicates extracts’ cytotoxicity towards the host cells.

Plant/Compound	Organ	Extract	Yields	*T. cruzi* Y Strain		*T. cruzi* Sylvio X10/1 Strain	
				Normalized Activity (%)	Average Cell Ratio	Normalized Activity (%)	Average Cell Ratio
*C. maritimum*	Stems	Decoction	29.2%	−1.42 ± 4.50	0.79 ± 0.05	−1.66 ± 5.74	1.36 ± 0.19
		Tincture	20.8%	68.1 ± 10.0	0.14 ± 0.06	70.2 ± 16.8	0.13 ± 0.01
		EOs	0.23%	0.00 ± 0.00	0.01 ± 0.01	55.7 ± 27.0	0.13 ± 0.11
	Leaves	Decoction	45.7%	13.6 ± 3.47	0.47 ± 0.05	11.8 ± 3.26	0.52 ± 0.23
		Tincture	26.5%	35.2 ± 4.07	0.26 ± 0.11	33.2 ± 12.2	0.38 ± 0.06
		EOs	0.30%	75.1 ± 13.1	0.02 ± 0.02	22.2 ± 18.9	0.02 ± 0.02
	Flowers	Decoction	37.8%	65.0 ± 6.04	0.73 ± 0.04	29.3 ± 0.69	2.00 ± 1.47
		Tincture	32.4%	35.4 ± 31.5	0.01 ± 0.00	73.3 ± 49.5	0.01 ± 0.00
		EOs	0.53%	107 ± 0.47	0.00 ± 0.00	12.0 ± 69.7	0.02 ± 0.02
*H. italicum* subsp. *picardii*	Flowers	Decoction	27.8%	−5.76 ± 1.51	0.12 ± 0.03	−10.6 ± 9.18	0.32 ± 0.07
		Tincture	32.6%	13.4 ± 8.36	0.09 ± 0.01	−7.68 ± 3.23	0.13 ± 0.01
		EOs	0.30%	76.1 ± 15.3	0.37 ± 0.14	93.4 ± 7.04	0.36 ± 0.24
Benznidazole ^a^				99.5 ± 0.45	1.82 ± 0.16	98.6 ± 0.62	2.11 ± 0.62

Data represent the mean ± SD of two independent experiments/each strain. ^a^ Positive control.

**Table 2:** Dose-response screening of anti-parasitic activity of the active extract, sea fennel flower decoction, against *T. cruzi* Y strain.

Extract/Compound	*T. cruzi* Y Strain			
	EC_50_^b^	Max. Activity (%) ^c^	CC_50_ (µg/mL) ^d^	SI ^e^
Active extract	17.7 ± 1.38 µg/mL	89.4	ND	>5.65
Benznidazole ^a^	3.97 ± 0.93 µM	100	ND	>101

Data represent the mean ± SD of two independent experiments. ND: not determined. ^a^ Positive control (3.97 µM = 1.03 µg/mL); ^b^ EC_50_ is a measure of potency; ^c^ Maximum activity is a measure of maximum efficacy against the parasite; ^d^ CC_50_ is a measure of cytotoxicity towards host cells; ^e^ SI indicates extract/compound selectivity towards the parasite.

**Table 3:** Dose-response screening of anti-parasitic activity of the active extract’s fractions **1** to **5** (hexane, dichloromethane, chloroform, ethyl acetate, water) against *T. cruzi* Y strain.

Extract/Compound	*T. cruzi* Y Strain			
	EC_50_ (µg/mL) ^b^	Max. Activity (%) ^c^	CC_50_ (µg/mL) ^d^	SI ^e^
Fraction **1**, Hex	0.47 ± 0.01	113	28.0 ± 0.90	59.6
Fraction **2**, Dcm	12.3 ± 0.35	97.0	79.3 *	>6.47
Fraction **3**, Clor	23.3 *	56.6	ND	>4.29
Fraction **4**, Acet	ND	39.4	ND	ND
Fraction **5**, H_2_O	ND	42.0	ND	ND
Benznidazole ^a^	0.92 ± 0.02	109	ND	>56

Data represent the mean ± SD of two independent experiments. ND: not determined. Hex: hexane, Dcm: dichloromethane, Clor: chloroform, Acet: ethyl acetate, H_2_O: water. ^a^ Positive control; ^b^ EC_50_ is a measure of potency; ^c^ Maximum activity is a measure of maximum efficacy against the parasite; ^d^ CC_50_ is a measure of cytotoxicity towards host cells; ^e^ SI indicates extract/compound selectivity towards the parasite. * Values obtained in one experiment (the second experiment did not display any significant toxicity against the host cells).

**Table 4:** Dose-response screening of anti-parasitic activity of falcarindiol against *T. cruzi* Y strain.

Compound	*T. cruzi* Y Strain			
	EC_50_ (µM) ^b^	Max. Activity (%) ^c^	CC_50_ (µM) ^d^	SI ^e^
Falcarindiol	6.8 ± 1.9	124	>100	>14.5
Benznidazole ^a^	26.8 ± 7.5	132	>400	>14.6

Data represent the mean ± SD of two independent experiments. ^a^ Positive control; ^b^ EC_50_ is a measure of potency; ^c^ Maximum activity is a measure of maximum efficacy against the parasite; ^d^ CC_50_ is a measure of cytotoxicity towards host cells; ^e^ SI indicates compound selectivity towards the parasite.

## Data Availability

The datasets generated during and/or analyzed during the current study are available from the corresponding author on reasonable request.

## Supplementary Materials

The following are available online at https://www.mdpi.com/article/10.3390/plants10112235/s1, Figure S1: Anti-parasitic activity of the active extract, sea fennel’s flowers decoction, and of the control benznidazole: dose-response curves normalized to infected and non-infected controls, Figure S2: Anti-parasitic activity of the active extract’s fractions and of the control benznidazole: dose-response curves normalized to infected and non-infected controls, Figure S3: ^13^C and ^1^H-^13^C 2D HMBC NMR spectra of the active and selective hexane fraction **1**, Table S1: Ligand ID Similarity, Table S2: Filtered resulting Parasitic Eukaryotic targets.
