# Supplementary material for: In Vitro Anti-Trypanosoma cruzi Activity of Halophytes from Southern Portugal Reloaded: A Special Focus on Sea Fennel (Crithmum maritimum L.)
Source: Plants (Basel). 2021 Oct 20;10(11):2235. doi: 10.3390/plants10112235 (PMC8625203; doi:10.3390/plants10112235)
Supplement: Supplementary file 1 [file plants-10-02235-s001.zip › plants-1434735-supplementary.pdf]

Supplementary material

Article

In Vitro Anti-*Trypanosoma cruzi* Activity of Halophytes from Southern Portugal Reloaded: A Special Focus on Sea Fennel (*Crithmum maritimum* L.)

Catarina G. Pereira [1], Carolina Borsoi Moraes [2,3], Caio H. Franco [2], Clarissa Feltrin [2], Raphaël Grougnet [4], Euzébio Guimarães Barbosa [5], Michele Panciera [6], Carlos Roque D. Correia [6], Maria João Rodrigues [1] and Luísa Custódio [1,*]

[1] Centre of Marine Sciences CCMAR, Faculty of Sciences and Technology, Ed. 7, Campus of Gambelas, University of Algarve, 8005-139 Faro, Portugal; cagpereira@ualg.pt (C.G.P.); mary_p@sapo.pt (M.J.R.)
[2] Department of Microbiology, Institute of Biomedical Sciences, University of Sao Paulo, Sao Paulo 05508-900, SP, Brazil; cbmoraes@unifesp.br (C.B.M.); caiohaddadfranco@gmail.com (C.H.F.); clarissaufsc@gmail.com (C.F.)
[3] Department of Pharmaceutical Sciences, Federal University of Sao Paulo, Diadema 09913-030, SP, Brazil
[4] Natural Products, Analysis, Synthesis, UMR CNRS 8038, Faculty of Pharmacy, University of Paris, 4 Avenue de l'Observatoire, 75006 Paris, France; raphael.grougnet@yahoo.fr
[5] Department of Pharmacy, Universidade Federal do Rio Grande do Norte, Natal 59064-720, RN, Brazil; euzebiogb@gmail.com
[6] Institute of Chemistry, State University of Campinas, Josue de Castro St., Campinas 13083-970, SP, Brazil; michele2707@hotmail.com (M.P.); roque@iqm.unicamp.br (C.R.D.C.)
* Correspondence: lcustodio@ualg.pt

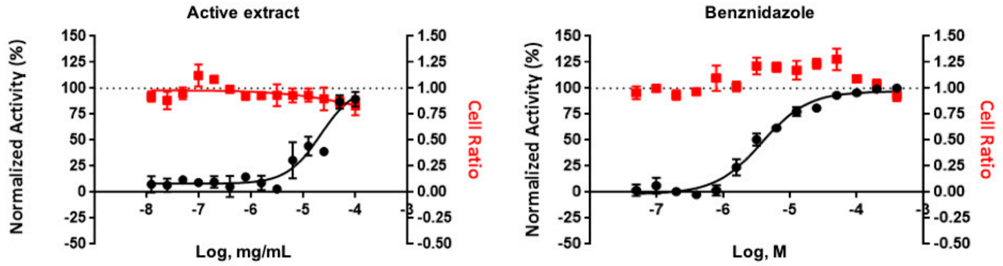

Figure S1. Anti-parasitic activity of the active extract, sea fennel's flowers decoction, and of the control benznidazole: dose-response curves normalized to infected and non-infected controls. Data refers to mean values of at least two independent experiments. Black dots refer to normalized activity while red dots refer to cell ratio.

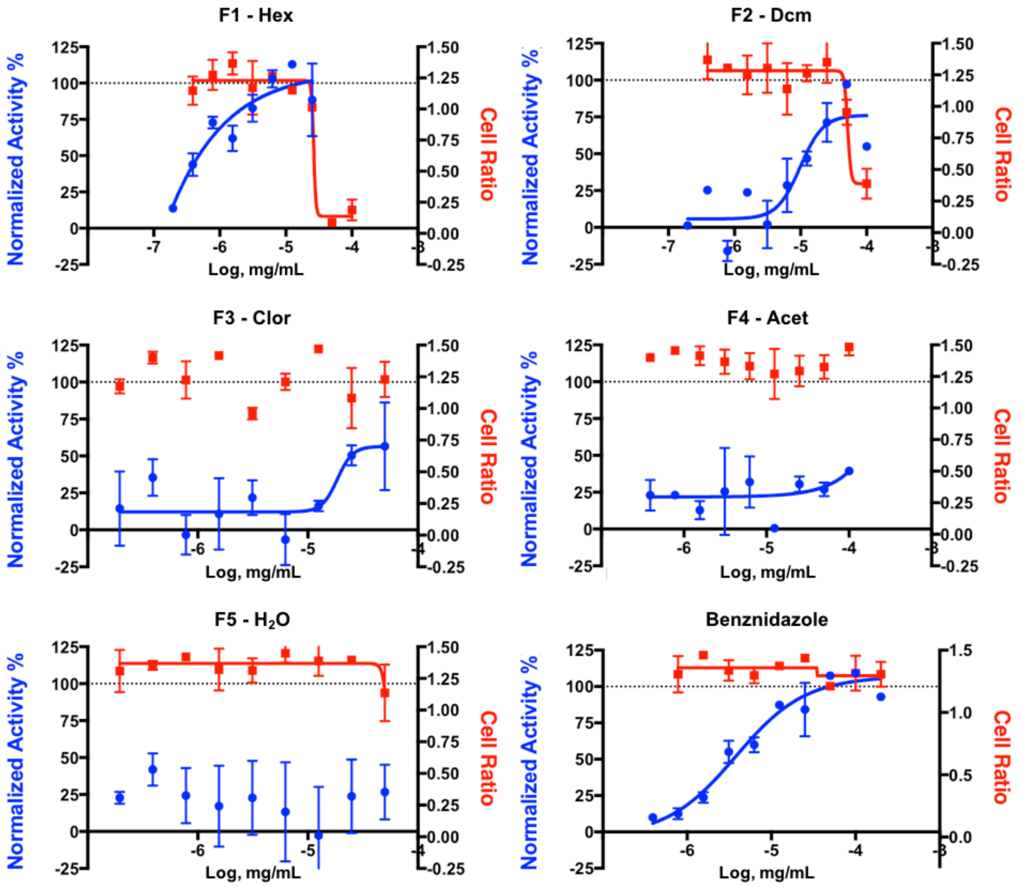

Figure S2. Anti-parasitic activity of the active extract's fractions (F1 - Hex, hexane; F2 - Dcm, dichloromethane; F3 - Clor, chloroform; F4 - Acet, ethyl acetate; F5 - H2O, water), and of the control benznidazole: dose-response curves normalized to infected and non-infected controls. Data refers to mean values of at least two independent experiments. Blue dots refer to normalized activity while red dots refer to cell ratio.

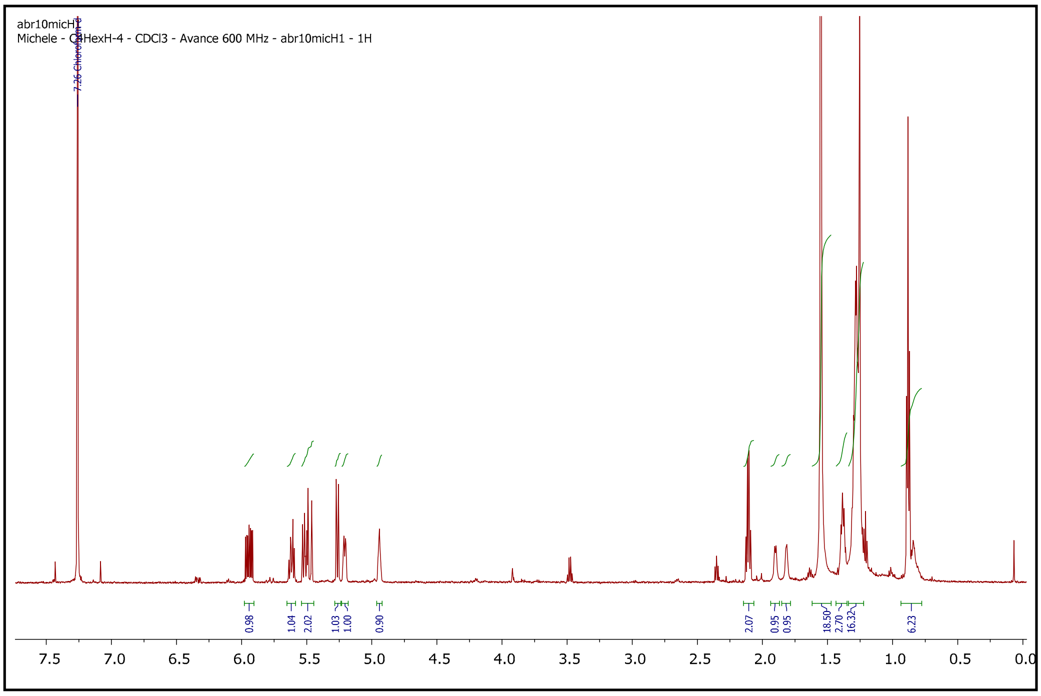
abr10micH1
Michele - C4HexH-4 - CDCl3 - Avance 600 MHz - abr10micH1 - 1H
7.26 Chloroform-d
0.98
1.04
2.02
1.03
1.00
0.90
2.07
0.95
0.95
18.50
2.70
16.32
6.23
7.5 7.0 6.5 6.0 5.5 5.0 4.5 4.0 3.5 3.0 2.5 2.0 1.5 1.0 0.5 0.0

(**a**)

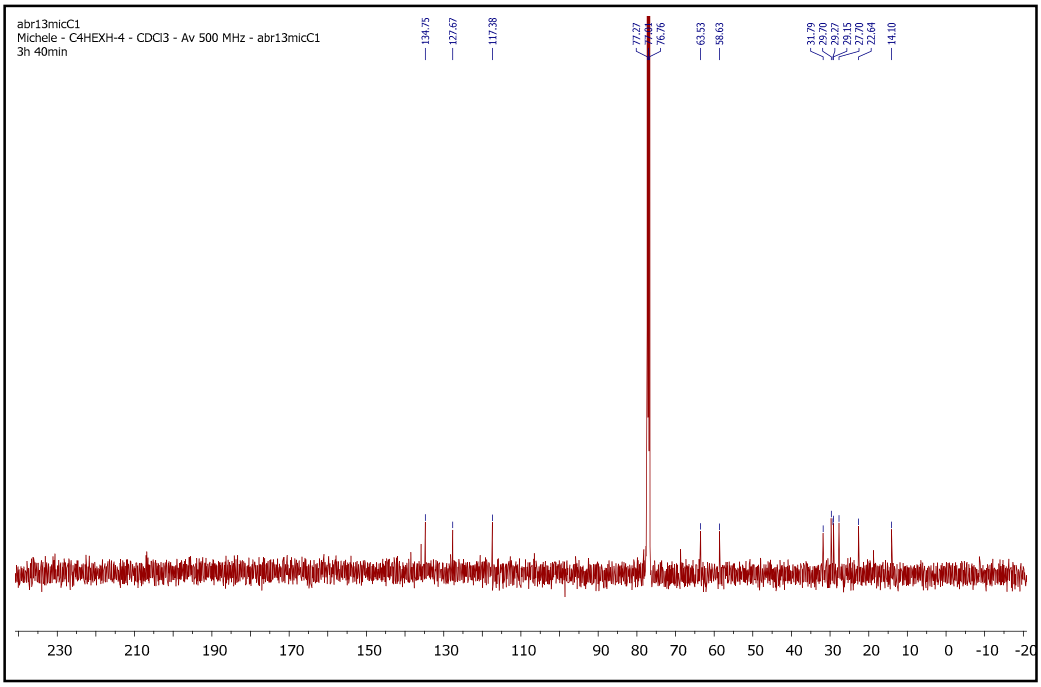
abr13micC1
Michele - C4HEXH-4 - CDCl3 - Av 500 MHz - abr13micC1
3h 40min
134.75
127.67
117.38
77.27
77.01
76.76
63.53
58.63
31.79
29.70
29.27
29.15
27.70
22.64
14.10
230 210 190 170 150 130 110 90 80 70 60 50 40 30 20 10 0 -10 -20

(**b**)

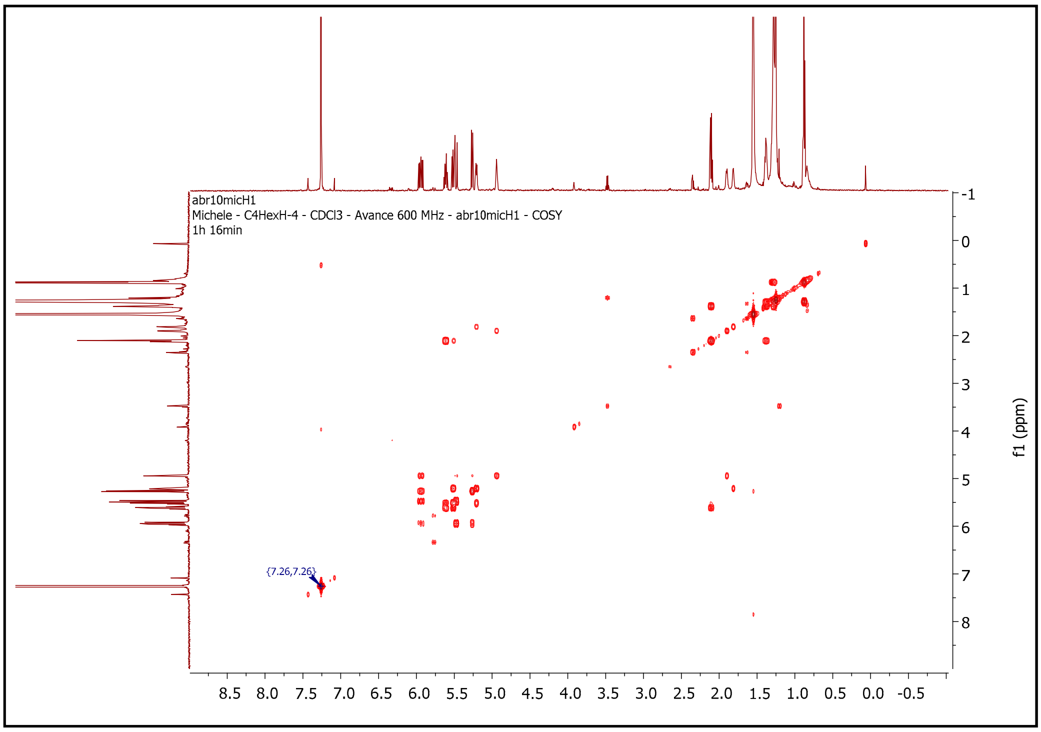

abr10micH1
Michele - C4HexH-4 - CDCl3 - Avance 600 MHz - abr10micH1 - COSY
1h 16min
(7.26,7.26)
f1 (ppm)

(c)

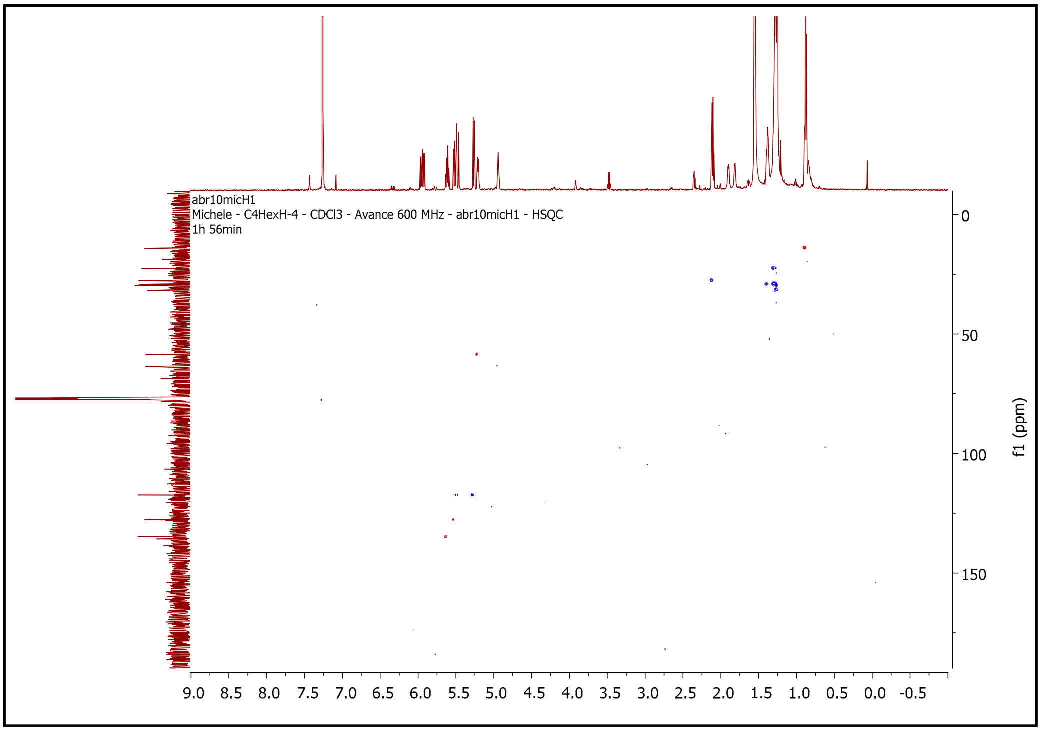

abr10micH1
Michele - C4HexH-4 - CDCl3 - Avance 600 MHz - abr10micH1 - HSQC
1h 56min
f1 (ppm)

(d)

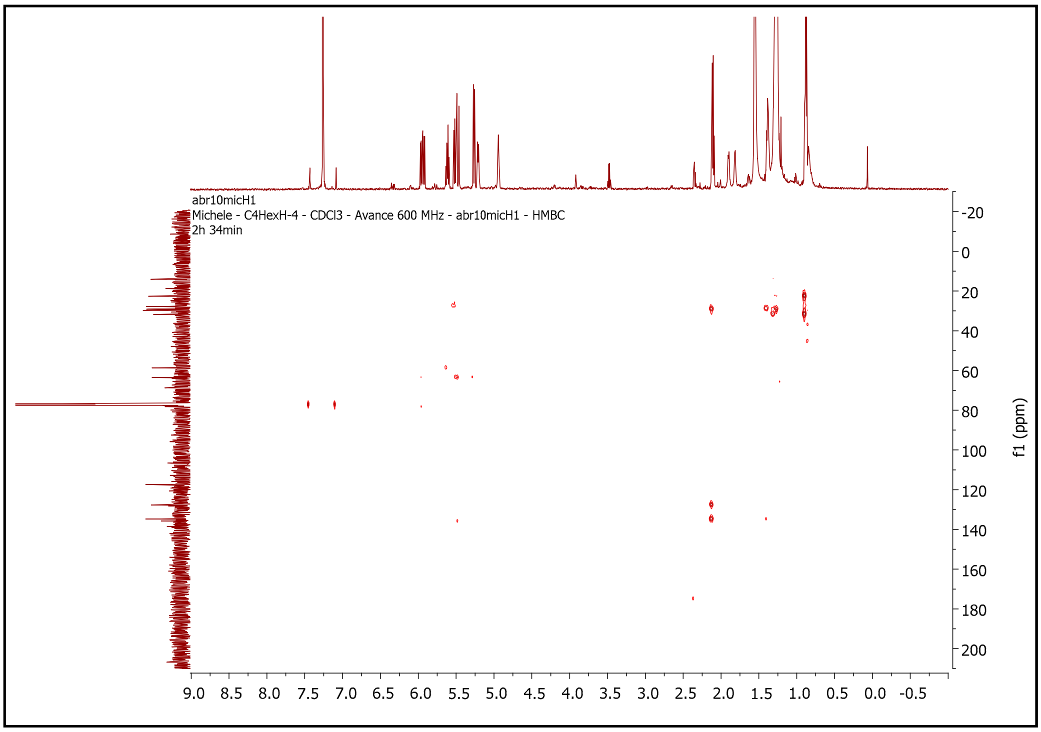

(**e**)

Figure S3. ^{13}C and ^{1}H-^{13}C 2D HMBC NMR spectra of the active and selective hexane fraction **1**. (**a**) Falcarindiol: ^{1}H NMR, $CDCl_3$, 600 MHZ; (**b**) Falcarindiol: ^{13}C NMR, $CDCl_3$, 125 MHZ; (**c**) Falcarindiol: ^{1}H-^{1}H 2D COSY NMR, $CDCl_3$, 600 MHZ; (**d**) Falcarindiol: ^{1}H-^{13}C 2D HSQCed NMR, $CDCl_3$, 600 MHZ; (**e**) Falcarindiol: ^{1}H-^{13}C 2D HMBC NMR, $CDCl_3$, 600 MHZ.

Table S1. Ligand ID Similarity.

4E6 0.904489; B9M 0.896859; OLI 0.894898; EPA 0.889473; LNL 0.888082; 13H 0.887077; C1J 0.885986; T65 0.883061; 7MS 0.876009; ZWI 0.875173; FAT 0.874765; BOM 0.874169; O12 0.863311; 8K6 0.862459; 10Y 0.862246; LDM 0.861636; 14Y 0.861298; 1EX 0.860092; PLY 0.859768; KKJ 0.858985; MUN 0.858948; HDS 0.858833; LTB 0.856802; 6G4 0.856686; LAX 0.856572; 7LE 0.856183; SPH 0.855778; HT5 0.854843; ODD 0.854031; 7E9 0.853703; LDA 0.853481; CAT 0.853404; 1WV 0.853378; 56S 0.852966; B0S 0.852606; XPM 0.852316; TER 0.851823; GTY 0.851466; L5P 0.851205; 9OH 0.850822; DSC 0.850362; OCD 0.84993; PTG 0.847643; C11 0.847453; ERU 0.847336; 3X1 0.847157; PN2 0.846272; D22 0.846143; PG6 0.846136; 6E0 0.846006; LIM 0.845947; 0GJ 0.845675; TNA 0.845641; TNT 0.845258; PG2 0.844952; 2RD 0.844677; B33 0.844434; 5A6 0.844221; B73 0.843532; IL2 0.843346; 7E8 0.843336; 1PG 0.842432; SSV 0.841515; 4HH 0.841461; TFR 0.841396; 9HO 0.841165; T24 0.840807; NDJ 0.840787; 1EY 0.84062; W01 0.84055; 5HE 0.840363; MYR 0.839795; LNA 0.839558; JAO 0.839186; STE 0.839113; 13S 0.838859; DRE 0.838415; ODT 0.83763; N10 0.83702; GER 0.837001; 6OB 0.836978; MYZ 0.836694; AFC 0.836689; MAY 0.836155; 4HX 0.83579; 11O 0.835657; 14V 0.835374; 9MN 0.835229; 3DP 0.834817; V10 0.834348; 12H 0.834199; NBA 0.834044; TRD 0.834028; EAH 0.833809; 2NV 0.83375; 2DE 0.833643; 140 0.833502; LSO 0.833446; ELD 0.833402; HJ2 0.833178; OLC 0.833054; KBP 0.832854; SPZ 0.829494; MHI 0.829219; BOB 0.828907; 2YG 0.828877; 4XQ 0.828161; MG8 0.827994; 3LA 0.827817; LA2 0.827697; 14U 0.827627; 2CF 0.825096; C15 0.825093; ARC 0.824747; 88P 0.824654; PLM 0.824256; SXV 0.82401; IEF 0.823879; 4M5 0.823478; VZZ 0.823404; AR0 0.823243; SP5 0.823236; 2YJ 0.823094; JRJ 0.823068; DJ3 0.822956; FJP 0.822952; 16A 0.822901; 1AG 0.822798; PET 0.822718; A55 0.822614; SZH 0.822592; FTT 0.822165; DME 0.82186; NER 0.821851; SCK 0.821749; 3PK 0.821553; 2JF 0.821466; 8E7 0.821428; TDA 0.821176; 1DO 0.821039; HO2 0.821038; Z6X 0.82093; 27Q 0.820918; PGF 0.820864; 5YL 0.820636; P15 0.820525; DPE 0.820516; K9H 0.820396; 4DY 0.820302; SXA 0.820064; ML5 0.82005; ELA 0.820003; OHM 0.819924; 6X4 0.819723; 6A4 0.819372; ETE 0.819311; 16P 0.819115; RTY 0.819029; 8XO 0.818513; SBY 0.818448; STB 0.81843; PJ2 0.818404; FFT 0.818205; JH3 0.818147; 298 0.817518; MFT 0.817507; DK3 0.817309; VCA 0.817136; MUP 0.817079; NKN 0.816512; AR2 0.815234; A5R 0.815191; 87O 0.81517; XLX 0.814952; FKS 0.814853; REC 0.814852; D12 0.814777; LLP 0.814673; 1QX 0.81444; XA2 0.814305; 0HH 0.813806; GRG 0.813703; F2P 0.813586; F23 0.813549; SKO 0.813278;

P3G 0.81311; O48 0.813077; VIO 0.813033; SQS 0.812941; 7K8 0.81271; 1K4 0.812444; D6M 0.81217; B9B 0.81202; N7E 0.811901; 5R7 0.811894; OCR 0.811864; N8E 0.811783; S1P 0.811713; GTS 0.811649; DAO 0.811636; CQR 0.811622; FPQ 0.811516; IVH 0.811465; P03 0.811194; FF8 0.810511; AS1 0.810445; 65D 0.810416; 4PS 0.810367; BUJ 0.810211; LHE 0.810202; 66F 0.8102; JAI 0.810131; TWN 0.810079; KLJ 0.810071; GZZ 0.81007; 1PE 0.810041; 5LF 0.810034; DPV 0.810003; 78M 0.809988; JSR 0.809907; 621 0.809183; MH9 0.80916; PN4 0.809102; YFD 0.809032; VAB 0.808929; 3M5 0.808785; UDT 0.808649; 1SV 0.808424; CLG 0.807108; 76V 0.8071; 1XG 0.807022; SC0 0.806988; 0LZ 0.806952; NPC 0.806907; 79N 0.806891; AI7 0.806846; HNC 0.806685; LYU 0.80665; HE0 0.806631; N1T 0.806414; 4WS 0.806386; EG2 0.80637; OP2 0.806244; PWZ 0.805767; F1K 0.805511; ARL 0.805496; J14 0.805453; PM5 0.805401; **SPM 0.805353**; 10A 0.805251; PO7 0.805172; HCY 0.805151; DP2 0.804884; D1I 0.80487; DQM 0.804868; 1E3 0.804852; PX9 0.804822; SQL 0.804642; 042 0.804582; ZBF 0.804171; LAU 0.804166; FHL 0.804156; R12 0.804064; 3MX 0.803958; 4R8 0.803819; P2E 0.803759; OQR 0.803714; SDR 0.803689; 8D7 0.803684; 2W2 0.803632; TE4 0.803491; AKS 0.803365; QLE 0.803351; EPM 0.803334; M7G 0.80331; O60 0.803308; 36J 0.803307; RNH 0.803001; T94 0.802994; CW2 0.802834; OPA 0.802818; ATI 0.802734; UFO 0.80268; 6IA 0.802545; DET 0.802539; 3VT 0.802514; 68C 0.802491; IFA 0.80242; MHC 0.80235; 0RV 0.800383; GEL 0.800382; 2TD 0.80029; OPD 0.80018; LEH 0.800091; OHN 0.800068; DFD 0.800013

Table S2. Filtered resulting Parasitic Eukaryotic targets.

3HD3; 4OFG; 4PHL; 2F6I; 7AOI; 4GF2; 6A6Y; 2O2Y; 1OKT; 6B5S; 6E0B; 3BJE; 2PSS; 2PT9; 2PT6; 2I7C; 3E1Z; 4BJK; 3AM3; 3AM5; 1UH5; 2AH2; 1NHG; 4Z09; 2M9H; 6I1N; 6HY1; 4UOE; 4BP3; 4BP1; 5NTG; 3BPF; 1FNT; 6B0S; 3RIE; 5L8Y; 5G2B; 5G57; 5L8C; 5L9H; 6RFW; 6RFN; 6RGK; 4JNO; 4B6M; 1NMR; 6FE3; 6QGU; 6QGP; 6B5L; 6B5M; 2GHU; 1Z7Q; 4CKU; 5TBO; 2KDN; 6B5R; 6B5T; 6B5N; 6B5P; 6B5O; 3NI8; 5DEL; 5JAZ; 1Q4J; 3P3K; 4Z22; 6YXS; 1TV5; 1I13; 2AAW; 6O24; 5NFR; 1VBJ; 1YHM; 1YHK; 2OUL; 3JQF; 1YHL; 4CWA; 2HTE; 4R1C; 4R1B; 4R19; 2Q8Z; 5K8S; 4MXR; 4Z0D; 4Z0E; 4Z0F; 3B7P; 6RZQ; 5K4T; 6SJQ; 1S29; 5G5V; 6RB6; 1HBK; 5K4W; 5K4V; 5K4Y; 5K50; 5K4Q; 5K4U; 3QS1; 3C02; 1PFZ; 3BPM; 4ZCQ; 3O8A; 3I65; 4AZ1; 3ENZ; 2B4G; 4J75; 4J76; 6RME; 6RNH; 6RMW; 1YVB; 6FDX; 6FRD; 6FTM; 2B4R; 3U3D; 3U31; 3V93; 6AXK; 4EU1; 3FR3; 3FR6; 3FRC; 3O6O; 5FI8; 5LGD; 1T2E; 1T2D; 1T2C; 1T25; 1T24; 3KQX; 3KQZ; 4R6T; 4R7M; 4K3N; 4X2T; 1AG1; 2C0D; 3FR9; 6I7D; 1NW4; 1Q1G; 2NQD; 3KR4; 3KR5; 3T8W; 2WWF; 2WWG; 5IFU; 1BW0; 6O26; 6LP1; 2VFE; 3E0U; 1MS1; 2NNR; 2Q0X; 5FSV; 5FSX; 6ZHI; 6MPV; 3H9U; 6SF9; 4R6W; 4R6X; 3UJA; 3UJC; 3UJD; 3UJ8; 3UJ9; 3UJ6; 3UJ7; 1SME; 4HHP; 3C3N; 6D24; 6D23; 4C0C; 3CVQ; 3F5M; 6GXQ; 2CIO; 2OZ2; 3UJB; 3DWC; 6OZU; 6UX6; 2J1Q; 6ZFW; 4E6Z; 3EOZ; 5ILQ; 3PNR; 5JOD; 6FDS; 6FDW; 6FV9; 5QTI; 5QTH; 5QTJ; 5QTE; 5QTD; 5QTG; 5QTF; 5MYP; 6SII; 2F2T; 2A0K; 2F67; 2F64; 2F62; 4MYL; 1YA7; 1YAU; 1YAR; 3HHI; 3DMT; 4E2B; 3IDS; 3INV; 3IRO; 6JKR; 6JKT; 6JL5; 2DJL; 2E68; 2E6A; 2E6F; 2E6D; 2WKF; 2W40; 4HNK; 4ZQT; 6FQZ; 6FQX; 4MW6; 4MW7; 4MVW; 4MVX; 4MW9; 4MVY; 4MW0; 4MW1; 4MW2; 4MW4; 4MW5; 4MWB; 4MWC; 4MWD; 4MWE; 3GUE; 4N7B; 4YA8; 4ZXG; 6LHJ; 4Y6M; 4W5B; 4W5C; 4FGZ; 6X44; 3BWK; 3BFK; 3N3M; 3S6B; 2PWP; 5ILN; 6TLB; 1XE5; 1XE6; 4MYK; 4MYN; 4MYF; 3MOR; 3CXG; 2X8L; 3BQK; 3BQL; 3BQI; 6KUC; 6KUB; 6KUD; 6X42; 4J57; 6RB5; 3AZA; 3AZB; 3AZ8; 3AZ9; 6D0X; 5AON; 3OZA; 3T60; 2RCY; 4IGE; 4IGF; 6SBR; 3JQ6; 3JQ8; 3JQ9; 3JQB; 3JQC; 3JQD; 3JQE; 3JQG; 3JQA; 3K21; 3EBH; 6JWV; 6KCK; 6KCM; 6KCL; 6KP7; 6KP2; 6KPR; 6FBA; 1LTK; 1XIV; 3JQ7; 3ZWZ; 5YIB; 5YIA; 5YIC; 5TPX; 5FUV; 5BK0; 6E62; 6E63; 6B08; 6AZM; 6AZZ; 5DYK; 6PHC; 6VLN; 3E7F; 3EB9; 4RX0; 5JNL; 5JO0; 5JBI; 5JC1; 5JMP; 4ORM; 4YS4; 4YY8; 3PEJ; 4NXJ; 3PR3; 3QKI; 6PBW; 6BQB; 3CS1; 6OHG; 2PU1; 2PU0; 2PTZ; 2PTY; 2PTX; 2PTW; 2H7W; 4PY6; 3TLX; 2M1H; 3ZG2; 3ZG3; 6RZY; 3JQR; 6S02; 3JQP; 3JQQ; 2Z8V; 2Z8W; 3K7Y; 4EOY; 6NL1; 4DSH; 3MMR; 1QNH; 1QNG; 6U9X; 6P5R; 2VSG; 1P19; 1P17; 2MXN; 6O2X; 6O7Z; 6O80; 6N3S; 2GPC; 1S0J; 1S0I; 1F3T; 4DSG; 1I0L; 1I0I; 1I14; 1P18; 4I15; 3M4U; 3ESF; 5L9A; 6O7Y; 1MXH; 1MXF; 1TCD; 2WYO; 3I05; 6JL3; 3TIK; 5LC1; 1XDN; 1XIY; 1N81; 2LKL; 1ZSO; 3J7A; 6OKK; 3UL3; 3IGM; 4EME; 2N3L; 2N7C; 1Z40; 2BPI; 3PSV; 3PSW; 3PVF; 2VFD; 2VFF; 2VFG; 2VFH; 2VFI; 3PWA; 3PY2; 6IEV; 4F1J; 4F1K; 3QRV; 2ZCG; 2PMN; 2PMO; 1A5C; 1D5C; 1LF2; 5NTF; 4QJB; 6DFK; 6RN1; 6RMO; 6II7; 3ULP; 4AOM; 3LRP; 4ZZ9; 5BMX; 5BMW; 5BNK; 5BRB; 1M7P; 1M7O; 1LYX; 1LZO; 6MB3; 6MHG; 1P9B; 1WOB; 1WOA; 1VGA; 1YWG; 3IBA; 3ICZ; 3ICM; 3ICN; 3ID0; 1V8B; 3SL0; 3SL1; 6RMD; 2MU9; 2MU8; 1PSM; 1II2; 1QXS; 2B4T; 3HBB; 4QY8; 3ICK; 1TC2; 4R3S; 6MUV; 6MUX; 5T5H; 3LMU; 5DKU; 5DKT; 5EZN; 6AQS; 6AQU; 1CEQ; 1CET; 1LEE; 4PG3; 3ZH2; 3LMT; 3LMV; 1VYQ; 2VFA; 6I7E; 5EZO; 4DPD; 1T26; 2WWH; 2WWI; 6I4J; 6I4I; 6I4L; 6I4K; 6I4D; 6I4F; 6I4E; 6I4H; 6I4G; 6UTH; 6UTG; 6UTJ; 6MUW; 6AXL; 7JGF; 7JGD; 7JGE; 3JBN; 3JBO; 3JBP; 3M1P; 5FSZ; 1AIM; 2MNI; 1U6F; 2FO8; 2A0M; 2AIM; 3KKU; 3OPZ; 4QH6; 4KLB; 3PJQ; 3I06; 3B69; 3LXS; 4LLR; 1F29; 1F2C; 1F2B; 1F2A; 1JVW; 1ME4; 1ME3; 1MR5; 1MS5; 1MS4; 1MS3; 1MS8; 1U9Q; 1YZV; 3K1O; 3KHM; 3KSW; 4H6O; 4JEQ; 2WUZ; 2WX2; 3Q37; 3ATY; 3ATZ; 4CK8; 4CK9; 4CKA; 4COH; 4UQH; 5AJR; 2Q2R; 6FMO; 4NEW; 2OMA; 6UEK; 1MS0; 1MS9; 1SUX; 1TC1; 1BZL; 1ML3; 2VUP; 5KLH; 2K8H; 1HXI; 2LTK; 3CBJ; 3CBK; 3CVP; 3H9D; 4AFV; 4AFP; 4AF8; 3DWV; 3DYF; 3DYG; 3DYH; 3EFQ; 3EGT; 5AEL; 5AFX; 5AHU; 2RM6; 2RM5; 1F2J; 5ZUH; 5FSU; 5FSY; 5NNO; 2K9X; 2J0E; 2B4V; 3JV1; 3K80; 3K81; 3K7U; 4G3J; 4G7G; 3OMU; 3P99; 3CV0; 3CVN; 3CVL; 4DK3; 4DK6;

4DKA; 4DNI; 2X2N; 2WV2; 3GW9; 3FZ0; 3G1Q; 1FX4; 1FX2; 2Q0G; 2Q0F; 2Q0E; 2Q0D; 2Q0C; 6DFT; 5I49; 5HZD; 5IDO; 5KAL; 16PK; 13PK; 2NOM; 2IKF; 2B56; 3JTL; 2WOI; 3IPM; 3STB; 2TOD; 6MD3; 1QU4; 3JRM; 3JSE; 2B51; 2VWA; 2FQ2; 2FQ0; 2LOE; 4O2X; 3K60; 3KNP; 3KO3; 3KO4; 3KO5; 3KNF; 3KOB; 3KOC; 3KO7; 3KO9; 4GM2; 4IOD; 4IRF; 4JLE; 4JUE; 3GYV; 3GYW; 3FS3; 3VUU; 3VUV; 4HJM; 3SUB; 3FNS; 3FNT; 3FNU; 2QAC; 2R9B; 2Q8B; 2Q8A; 5LG9; 5LM3; 5LLT; 5T3N; 5T8U; 4C81; 4C82; 5KBF; 4M1N; 4MZB; 4MZC; 4O32; 2OFN; 1TQX; 2FU0; 1YXE; 1Y6Z; 3GZL; 3GZM; 2BBX; 3KOD; 4GFT; 3NTJ; 4R1E; 4QT2; 4QT3; 4J4N; 4JFA; 4K2U; 3R3J; 4ADS; 2VN1; 2W41; 4CXM; 3TGH; 3CH2; 3CH3; 3VGJ; 4TR9; 4TWA; 5CFI; 5CFJ; 4XBI; 4WIN; 4WIO; 4WIM; 5J61; 5JLD; 5CS2; 4MZL; 2OK8; 2H2Y; 1RL4; 2FOI; 1Z6G; 2C07; 4P7S; 4P7M; 1HN6; 4J56; 4YDQ; 2YOG; 2YOH; 6FQY; 5XVU; 6HCV; 6HCU; 5JWC; 5JWB; 2OK7; 3CPZ; 2XF1; 2WBF; 3C64; 3CML; 2ZA1; 2ZA2; 2ZA3; 2YK0; 2Y8D; 3F9Q; 3QVC; 2OP1; 2OOS; 2PC4; 5ZNC; 5ZNI; 5BWY; 6DSR; 6DSQ; 6DSS; 6O25; 6O28; 6O2A; 6O29; 5X7V; 6S8T; 6N7Q; 6N87; 2NQ8; 2OKI; 2OKH; 2OL4; 1V0B; 1V0P; 1V0O; 2JKF; 2EPH; 1OB3; 1PA3; 1LF3; 2A94; 1RY6; 2BJU; 1YDV; 3I68; 3I6R; 2YOF; 4PLZ; 4B1B; 2WE6; 4D60; 3QVI; 2OP0; 6LEZ; 6LEV; 6LEU; 6LHI; 6LH9; 5E16; 5MI0; 6O2B; 6O2C; 4OFF; 2JKG; 2H1R; 1O5X; 2FBN; 1JYM; 1LDG; 1LS5; 1RQC; 1ZHG; 1Z6B; 4MZJ; 4MZK; 4V3E; 1ONF; 2ABW; 5TBD; 4CQA; 4CQ8; 4CQ9; 3H79; 6NEY; 6ZPM; 4PI3; 4DS2; 1EWP; 1EWO; 1EWM; 1EWL; 6HYQ; 6HYI; 5KGQ; 4XUI; 5CUX; 5CUV; 5CUU; 6NIM; 6NP7; 5QPM; 5QPL; 5QPO; 5QQ0; 5QPN; 5QPQ; 5QQ2; 5QPP; 5QQ1; 5QPS; 5QQ4; 5QPR; 5QQ3; 5QPE; 5QPD; 5QPG; 5QPF; 5QPI; 5QPH; 5QPK; 5QPJ; 5QQB; 5QQA; 5QQC; 5QPU; 5QQ6; 5QPT; 5QQ5; 5QPW; 5QQ8; 5QPV; 5QQ7; 5QPY; 5QPX; 5QQ9; 5QPZ; 6R0A; 6R0B; 6R05; 6R04; 6R07; 6R06; 6R09; 6R08; 6SDP; 6SDO; 6SDQ; 6SE2; 6SDN; 6SF8; 6SI5; 6SHV; 1OGL; 1XO7; 4QFH; 4E1E; 4E2D; 4DWB; 3V94; 4BY0; 3IRM; 3IRN; 3LC0; 3QV9; 6JKQ; 5AQ1; 4DWG; 4DXJ; 2V5B; 6V6H; 1OGK; 1W61; 3HRK; 4UVR; 2QAE; 6JL4; 6JL6; 4C27; 4C28; 4DZW; 6SFA; 6T7N; 1CI1; 1XQ7; 2DJX; 3IUT; 3KJS; 3PRV; 3CLB; 1K3T; 1GXF; 2H2Q; 4U0Q; 3CL9; 5MVV; 4RXC; 4RXE; 4RYP; 5QTK; 5QTA; 5QTC; 5QTB; 5QT9; 5QT8; 4BP8; 5VTL; 5QT5; 5QT4; 5QT7; 5QT6; 6R36; 6R38; 6R37; 6R39; 6T4R; 6T68; 2MYG; 2OGD; 2LJ8; 4DK4; 4DK2; 4DL8; 4DKB; 4DLC; 3HJ4; 3HIY; 3I3G; 3HRI; 3BNW; 4RXD; 2YHU; 3LSQ; 3FB3; 2P1C; 6E4N; 6E4P; 6E4O; 4BP9; 1GY8; 2LJ4; 2I19; 2EWG; 2X0S; 3HJ1; 2W7T; 2YHI; 3LSS; 3F9R; 4BQH; 2HIG; 2HKE; 2CNB; 1PGJ; 2AMH; 5MZA; 3OTX; 3QD9; 3QCP; 2QJC; 4CBU; 6S8U; 6XMJ; 2WDT; 6SOZ; 6SOY; 3T64; 2MUF; 5JKQ; 5WRU; 2MYF; 2MU6; 2MTY; 3P1W; 3PEH; 3FKM; 2QU8; 2R77; 2PBF; 6TJ4; 6TJ3; 5VS7; 5ULC; 2MMO; 2MMN; 6YXT; 2F8M; 1SYR; 3KHD; 3KKK; 4N10; 4N11; 4N0Z; 4QNS; 2WAU; 3FOW; 3MWA; 3IED; 3N34; 3N2M; 4ZCR; 4ZCS; 4ZCT; 3LLT; 3FI8; 2R0J; 2ONU; 2PLW; 2Q0V; 2Q8L; 6SY0; 5WOF; 4NBI; 4NBJ; 1IUE; 6SBQ; 2MRC; 1SQ6; 2BSX; 1YJ8; 2F84; 2MU7; 3OZ7; 3BAR; 3T70; 3T6Y; 3UOW; 4ZEW; 4ZEX; 4ZEV; 2Y8C; 3E95; 3S9Y; 2QAF; 6KSH; 5JQK; 5FOC; 5FOD; 4NCX; 4OLF; 6SU9; 6T7K; 1XIQ; 6ZN3; 4HQF; 4HQK; 3SRJ; 3SRI; 3VDJ; 3VDK; 3VDL; 6UC5; 5TIK; 6H5N; 3JWP; 4Q15; 4H02; 4WI1; 6GJG; 4Y6R; 4Y6S; 5JR6; 4Y6P; 4Y67; 5JMW; 3SFK; 4O6Z; 6VTN; 6VTY; 2RJI; 2MSA; 5BOO; 4WAT; 5YID; 5YIE; 5TIH; 6RCU; 5VR5; 1CEJ; 2IGY; 2IGX; 6E11; 6E10; 5JWA; 3N72; 2P65; 1ME6; 6T4D; 6SSZ; 1M43; 1Y13; 1XQ9; 1W6I; 1W6H; 4FYM; 3PHC; 3AM4; 3AUA; 3AU9; 3MSE; 3NIE; 4D73; 3BPW; 3CLV; 4B7U; 4Z0S; 4Z0J; 4YWI; 4YXG; 4ZCP; 3LT4; 3LSY; 3LT0; 3LT1; 3LT2; 6JW9; 6CA8; 5GZP; 5GV4; 6R8G; 2LU1; 1U4O; 1U5C; 1U5A; 1U4S; 6WFW; 1ZSN; 1ZW1; 1ZXB; 1ZXL; 2BMA; 3UM6; 3UM8; 3VI2; 2PML; 6JWS; 6A2P; 6A2O; 6A2L; 6A2K; 6A2N; 6A2M; 6KOT; 5FO4; 6HL7; 7BT5; 1NNU; 4LVN; 4LVO; 4QEX; 4U1G; 4U0R; 6D01; 6D11; 6B0H; 6O23; 6PHF; 6PHB; 6PHD; 6ULF; 6ULE; 1LF4; 1ZRO; 1ZRL; 6ZDX; 3JSU; 3DGA; 3DG8; 3UM5; 3AU8; 3QG2; 3QGT; 6JWT; 6JWU; 6JWX; 6JWW; 6JWZ; 6JWY; 6JWR; 6JWQ; 6I55; 1J3K; 1J3J; 1J3I; 1V35; 1VRW; 6I4B; 6W00; 1CJB; 6RCV; 6RW3; 2MUD; 2J5L; 6PBV; 5FMG; 1OB1; 3J79
